# A simple and rapid technique of template preparation for PCR

**DOI:** 10.3389/fmicb.2022.1024827

**Published:** 2022-11-10

**Authors:** Yunyun Liu, Jia Chen, Yi Cheng, Yi Li, Xinwen Li, Zhengbing Zhang, Xiumei Xu, Yufeng Lin, Jianping Xu, Zhimin Li

**Affiliations:** ^1^Institute of Bast Fiber Crops and Center of Southern Economic Crops, Chinese Academy of Agricultural Sciences, Changsha, China; ^2^Plant Protection and Inspection Station, Agriculture and Rural Department of Hunan Province, Changsha, China; ^3^Department of Biology, McMaster University, Hamilton, ON, Canada

**Keywords:** simple PCR template preparation, microorganism, potassium hydroxide, long fragment amplification, high through-put PCR

## Abstract

Many techniques have been developed for extracting DNA, but most are often complex, time-consuming, and/or expensive. In this study, we describe a simple, rapid and cost-effective technique for preparing DNA template for PCR. This technique involves 0.1 M potassium hydroxide treatment at 100°C for 10 min followed by centrifugation. The suspended centrifuged sediments were shown as excellent templates for PCR. Templates prepared using this technique worked for diverse microorganisms belonging to bacteria, fungi and oomycetes and their amplification efficiencies were comparable to/better than those prepared using common but relatively more complex, time-consuming, and/or expensive methods, including commercial DNA extraction kits. Furthermore, this technology is suitable for high-throughput batch processing and for amplifications of long DNA fragments. Flow cytometry and scanning electronic microscopy analyzes showed that this technique generated primarily damaged cells and cell-bound DNA, not free naked DNA. This technique provides a great convenience for simple PCR template preparation.

## Introduction

The polymerase chain reaction (PCR) is one of the most widely used techniques in molecular biology. Preparation of template DNA is a critical step in PCR. Since the middle of the last century (Brown and Watson, 1953), many protocols have been developed to extract DNA from a variety of biological samples. However, there are continuous demands for high throughput, rapid, and cost-effective methods. In microbiology research, PCR is commonly used for a variety of purposes, often involving testing many samples that include organisms belonging to different species, Genera, Families, Orders, Classes, Phyla, Kingdom, and Domain. In many cases, a simple, rapid and widely applicable technique for DNA template preparation would be of particular interest.

Most microorganisms have a cell wall and different groups of microbes have cell walls composed of different macromolecules that may change during their development. Cell wall represents a significant barrier in DNA extraction in all microorganisms. To effectively extract DNA from cells, enzymes and/or physical methods are typically used to first break the cell wall and release the intracellular contents, including DNA. For example, cell wall degrading enzymes such as lyticase are commonly used in fungal DNA extraction methods to remove the fungal cell wall (Xu et al., 1994, 2000). However, the cell walls of some fungal species are recalcitrant to degradation by some enzymes, due to most enzymes can only degrade some specific components of fungal cell walls. Similarly, certain bacteria also contain tough cell walls that are difficult to break and extract DNA from within. For those organisms, the alternative often involves deep freezing samples in liquid nitrogen followed by mechanical force grinding using mortar and pestle to break the cells. After cell breakage, the remaining steps are commonly shared among conventional protocols of DNA isolation, including removing other cell components such as proteins (especially DNA degrading enzymes), membrane lipid bilayer and so on, by phase separation using chloroform and/or phenol (Lecellier and Silar, 1994; Harju et al., 2004), before the extracted DNA samples are used as templates for downstream work. While relatively inexpensive, physical method for breaking cells using liquid nitrogen and grinding with mortar and pestle usually requires a larger quantity/volume of samples to obtain sufficient DNA. In contrast, while less physically demanding, enzymatic hydrolysis is typically more expensive. Regardless the cell wall-breakage methods, most current protocols for DNA extraction are relatively time-consuming and not conducive for high-throughput applications.

To simplify template DNA preparation for PCR amplifications, several modifications have been made. Some of these modifications involve chemical agents such as sodium dodecyl sulfate (SDS) or sodium hydroxide (NaOH) solutions to break cell walls (Cenis, 1992; Akada et al., 2000; Truett et al., 2000; Vingataramin and Frost, 2015). SDS is an ionic detergent with two inert ends, a hydrophobic twelve alkyl at one end and an ionizade sulfate radical at the other. SDS has relatively stable chemical properties in water, even in boiling water. However, as a surface-active agent, SDS can only break the weak interaction forces such as van der Waals force and hydrogen bonds, but not covalent bonds (Kokufuta et al., 1998; Zhan et al., 2020). Specifically, SDS can only denature the lipid bilayer structure of cell membrane and the protein tertiary structure by breaking weak interaction forces. However, it cannot break the covalent bonds of polysaccharides or peptidoglycans which compose the cell wall of fungi and bacteria, respectively, (Sørensen et al., 1990; Quiberoni et al., 2000). In contrast, due to their relatively active chemical properties, alkali agents such as sodium hydroxide can help break covalent bonds in cell walls (Freytag and Mendgen, 1991; Hartley and Morrison, 1991).

In the past few decades, many studies have shown that alkali (primarily sodium hydroxide) solutions can efficiently degrade polysaccharides such as cellulose, hemicellulose, pectin and so on, and higher temperature can facilitate such reactions (Whistler and BeMiller, 1958; Krochta et al., 1988; Ouajai and Shanks, 2005). Indeed, glycosidic bonds are relatively easily broken in alkali solutions (Knill and Kennedy, 2003; Glaus and Van Loon, 2008). In addition, the lipid bilayer structure of cell membrane can also be destroyed in an alkali solution by a chemical reaction called saponification (Körner et al., 1992; Greenwood et al., 2016). Thus, theoretically, both cell wall and cell membrane could be broken down by chemical reactions using an alkali agent. However, strong alkali solutions (pH > 9) can also cause DNA denaturation (from double stranded to single stranded) and depurination (Chamsart et al., 2001). While denaturation is necessary for PCR, depurination is not desirable and should be minimized. Thus, it’s necessary to identify an alkali solution that can break down cell wall and cell membrane but maintain DNA sequence integrity. Indeed, sodium hydroxide solution has been included in several DNA rapid-extraction protocols (Truett et al., 2000; Vingataramin and Frost, 2015).

How to precipitate DNA molecules released from cell wall and cell membrane-lysing solutions is another key step in most DNA preparation procedure. Alcohols have both electrostatic and structural effects on DNA molecules that can condense DNA into compact structures (Arscott et al., 1995; Bloomfield, 1996). The compacted DNA molecules are then precipitated by high centrifugal forces. Ethanol or isopropanol which can be easily removed, is commonly used for DNA precipitation from a solution (Michaels et al., 1994; Freitas et al., 2006). Cells treated by an alkali solution and DNA precipitated by alcohols are commonly carried out independently as two steps during DNA extraction procedures. However, one protocol named EtNa (Et for ethanol and Na for NaOH) showed that the two steps could be combined into one for DNA template preparation, yielding templates suitable for PCR for Gram-negative and Gram-positive bacteria and for yeasts (Vingataramin and Frost, 2015). However, there is a significant drawback in this combined protocol. Specifically, the azeotropic temperature of ethanol and water is 78.3°C but to break the cell wall of most microbial species, a higher temperature treatment (e.g., water bath at ≥80°C) is required using the EtNa extraction protocol. Consequently, if this protocol is used, the caps of tubes containing the mixture cannot remain closed when placed in ≥80°C water bath and will explode to open, causing health hazards and potential contamination among samples. Consequently, the EtNa protocol is not commonly used for many species that require a high temperature treatment to break cell wall.

Here, we develop an alternative and highly efficient protocol to eliminates these problems. Compared to other rapid techniques, our protocol can be applied to a broad range of microorganisms, in our case a total of 15 species representing 13 families, 6 phyla, and 3 Kingdoms/Domains. This protocol uses a combination of potassium hydroxide + boiling (100°C) + centrifugation (PBC) treatments. Further experiments showed that the end products of this treatment were not naked free DNA, but damaged cells. Compared to SDS-treatment, sodium hydroxide boiling, and EtNa methods, PBC is simpler, easier to operate, and can be applied to a wider group of organisms. PCR using the obtained templates resulted in PCR amplification qualities similar to those using templates prepared using commercial DNA extraction kits.

## Materials and methods

### Microbial species and microbial sample preparations

#### Microbial species used in this study

To ensure the broad applicability of our new DNA extraction method to different groups of organisms, strains from 15 microbial species were selected for this study. These 15 species belonged to 13 families, 6 phyla, and 3 Kingdoms/Domains. The details of strain names and their taxonomic representations are shown in Table 1. Most of these species are commonly used in microbiological research and for teaching purposes.

**Table 1:** Microbial species and strains (in parenthesis) analyzed in this study.

	Species (strain name)	Family (order)	Phylum	Domain/Kingdom
1	*Escherichia coli* (DH5α)	*Enterobacteriaceae*	*Proteobacteria*	Bacteria
2	*Bacillus thuringiensis* (YH-C)	*Bacillaceae*	*Fibrobacteres*	
3	*Saccharomyces cerevisiae* (BY4741)	*Saccharomycetaceae*	*Ascomycota*	Fungi
4	*Scheffersomyces stipitis* (CBS6054)	*Debaryomycetaceae*		
5	*Fusarium verticillioides* (YLDL)	*Nectriaceae*		
6	*Fusarium proliferatum* (H10L)			
7	*Aspergillus niger* (7806FL)	*Aspergillaceae*		
8	*Cladosporium tenuissimum* (zc03)	*Cladosporiaceae*		
9	*Alternaria alternate* (glx1)	*Pleosporaceae*		
10	*Moesziomyces antarcticus* (BDH2-1)	*Ustilaginaceae*	*Basidiomycota*	
11	*Ustilago maydis* (SG200)			
12	*Agrocybe aegerita* (wl01)	*Bolbitiaceae*		
13	*Pleurotus geesteranus* (y58)	*Pleurotaceae*		
14	*Phytopythium vexans*(HF1)	*Phytopythium*	*Pythiales*	Oomycota
15	*Phytophthora capsici* (Leonian)	*Peronosporaceae*	*Peronosporales*	

All microbial stains above are conserved in Institute of Bast Fiber Crops, CAAS.

#### Microbial growth

The microbial strains were cultured following standard laboratory conditions used for each species. Briefly, *Escherichia coli* DH5α was cultured in Luria-Bertani liquid medium (LB medium, containing tryptone 10 g/l, yeast extract 5 g/l, NaCl 10 g/l) at 37°C in a rotary shaker (180 rpm) overnight. *Bacillus thuringiensis* (isolate YH-C), *Moesziomyces antarcticus* (BDH2-1) and *Ustilago maydis* (SG200) were cultured in YEPS light liquid medium (10 g/l Yeast Extract, 4 g/l Peptone, 4 g/l Sucrose). *Saccharomyces cerevisiae* (YB4741) and *Scheffersomyces stipites* (CBS 6054) were cultured in yeast extract peptone dextrose (YPD, 10 g/l Yeast Extract, 20 g/l Peptone, 20 g/l Dextrose) liquid medium, and were incubated in a shaker incubator at 30°C at a rotating speed of 180 rpm overnight. Mycelia of four filamentous ascomycete fungi *Fusarium verticillioides* (strain YLDL), *Fusarium proliferatum* (strain H10L), *Aspergillus niger* (strain 7806FL) and *Cladosporium tenuissimum* (isolate zc03) were obtained by culturing on YEPS light solid agar (add 15 g/l agar) medium in a 28°C incubator. Finally, tissue cultures of two cultivated mushrooms *Pleurotus geesteranus* (strain y58) and *Agrocybe aegerita* (strain wl01), and mycelial cultures of two plant pathogenic oomycetes *Phytopythium vexans* (strain ZJJ-cn) and *Phytophthora capsici* (strain Leonian), and the common plant fungal pathogen *Alternaria alternata* (strain glx1) were cultured on PDA solid plates kept in a 28°C incubator for up to a week until sufficient mycelia were grown.

#### Microbial sample collection and preparation

To compare the efficiencies of various DNA extraction protocols for DNA template preparations for PCR, the microbial cells were harvested as uniformly as possible. Briefly, microbial samples cultured in liquid media were all collected by centrifugation at 13000 ×*g* for 1 min and washed with an equal volume of sterile ddH_2_O for 3 times. The pelleted cells were then resuspended in sterile ddH_2_O and adjusted to an optical density corresponding to about 10^7^ cells/ml for fungi by an Automated Cell Counter (TC20, Bio-Rad) and OD_600_ = 0.6 for bacteria by a microplate reader (infinite M200PRO, TECAN). The suspended cells were then divided into 100 μl aliquots, with each aliquot to be used for a different DNA extraction protocol. Fungal mycelial samples collected from solid plates also were divided into several equal aliquots, with each aliquot suspended in 100 μl sterile ddH_2_O.

To test the sensitivity of our DNA template preparation protocol to cell density, the vegetative cells or spores of three species were used in this experiment. The three species were all fungi, *S. cerevisiae, M. antarcticu*s, and *A. niger*. For *S. cerevisiae* and *M. antarcticu*s, actively growing vegetative cells in liquid cultures were used. While for *A. niger*, their conidia spores were collected from actively growing cultures on solid medium plates using 0.03% Tween-80 solution. The vegetative cells and spores were serially diluted with sterile distilled water and counted by the Automated Cell Counter (TC20, Bio-Rad). Cell numbers of 10 cells/ml to 10^8^ cells/ml were used in our analyzes.

### DNA extraction and template preparation protocols

EtNa is a very simple protocol of genomic DNA extraction for bacteria and yeast (Vingataramin and Frost, 2015), and our PBC method was developed based on it. During the development of PBC, to compare the treatment effects, samples treated by EtNa as controls were conducted according to the protocol described in the original literature (Vingataramin and Frost, 2015).

#### Development of PBC

##### Alcohol selection and concentration optimization

Ethanol, isopropanol, and n-propanol have all been used for DNA precipitation (Gissmann et al., 1982; Michaels et al., 1994; Freitas et al., 2006). The azeotropic temperature of ethanol/water, isopropanol/water and n-propanol/water are 78.07°C (EtNa), 80.33°C and 87.81°C (query on^1^), respectively. To overcome the deficiency of low azeotropic point in the EtNa protocol, we decided to test the effectiveness of various concentrations of n-propanol for DNA precipitation to avoid the problem of caps opening while placed in a hot water bath at 80°C.

To test the effectiveness of n-propanol for precipitating DNA, in the alternative protocol, we changed the ethanol in EtNa lysing buffer into n-propanol, and the other components (NaOH and EDTA) remained unchanged. Two fungi *S. cerevisiae* and *M. antarcticus* were used in this test. Specifically, the fungal cell suspensions were mixed with lysing buffer, and the final mixture contained 0.2 M NaOH (The same concentration as in the original EtNa protocol), 2.25 mM EDTA, and different n-propanol concentrations of 40, 30, 20, 10% and 0(v/v) in different treatments, respectively. [It was previously reported that about 33.3% n-propanol could precipitate DNA (Gissmann et al., 1982), thus we set the n-propanol concentration gradient from 10 to 40%.] Then the mixture was heated at 85°C for 10 min. For the original EtNa protocol, the mixture contained a final concentration of 61% ethanol and heated at 80°C for 10 min as a control (identical to that described in the original protocol). And then the mixtures were centrifuged at 13000 ×*g* for 10 min. After removing the final supernatants, the centrifuged sediments were dried at room temperature to volatilize alcohol and dissolved in 100 μl sterile water.

##### Alkali selection and concentration optimization

Potassium hydroxide (KOH), sodium hydroxide (NaOH) and lithium hydroxide (LiOH) are all strong alkaline agents (Das and Barringer, 2006; Max et al., 2009). Here we compared the treatment effects of these three alkalis on fungal DNA extraction. Cell suspensions of three fungi *S. cerevisiae*, *M. antarcticus* and *A. niger* were used in this experiment. To compare the treatment effects of different alkali agents, all three alkalis were set at the same concentration of 0.2 M in the final mixture (alkali solution mixed with cell suspension). No alcohol nor EDTA was used in this experiment. Since the mixture did not contain any alcohol, the water bath temperature was set at 100°C for 10 min.

After KOH was identified as the best among the three alkaline agents for PCR template preparation (please see results below), we conducted a concentration optimization experiment. KOH was set at a series of gradient concentrations of 0.05 M, 0.1 M, 0.2 M, 0.3 M, 0.4 M and 0.5 M in the final mixture (KOH solution mixed with cell suspension), respectively. These mixtures were treated in a 100°C water bath for 10 min.

All the mixtures were centrifuged at 13000 ×*g* for 10 min after the water bath. After removing the supernatant, the sediments were, respectively, dissolved in 100 μl sterile distilled water before PCR.

##### PBC (potassium hydroxide + boiling + centrifugation) protocol

After the optimization of alcohol and alkali conditions as described above, the finalized PBC method was determined. In the PBC protocol, the final mixture contains KOH at a concentration of 0.1 M (without any other additional chemical), and then incubates at 100°C water bath for 10 min. During of the development of PBC, all mixtures were centrifuged at 13000 ×*g* for 10 min after treated with hot water bath, and then the supernatants were removed. The centrifuged sediments were each resuspended in 100 μl sterile water, and then used in PCR.

##### Comparison with other protocols for PCR template DNA preparation

We compared the effectiveness of PBC with four other rapid DNA preparation methods. Two methods used SDS and NaOH, respectively, as the main reactant to remove cell wall, and we followed the specific protocols described in the relevant literatures, i.e., the TE/SDS protocol in Cenis (1992) and the HotNaOH protocol in Truett et al. (2000) respectively. The remaining two protocols were those described in the Fungus Genomic kit (Aidlab Biotechnology) and Bacteria Genomic DNA Kit (CW Biotech) and they were used to extract DNA from fungal and bacterial samples according to the manufacturers’ instructions, respectively. Altogether, 15 species of microorganisms were used in this comparative experiment (Table 1). To compare the DNA templates prepared using different techniques, all the initial cell densities were standardized to the same concentration and all the final centrifuged sediments were dissolved in 100 μl sterile water.

In this study, all experiments of DNA template preparation and PCR were repeated at least twice for each treatment. Consistent results among repeats were obtained.

### Genes and primers used in this study

To assess the effectiveness of prepared DNA as templates for PCR, we used the following gene fragments. For prokaryotes, part of the 16S rRNA gene was chosen for testing using primers F27 and R1494. For fungal and oomycete strains, the consensus barcode locus, the internal transcribed spacer (ITS) regions of the ribosomal RNA gene cluster, was tested using primers ITS1 and ITS4. Both the 16S rRNA and the ITS DNA fragments were multicopy and relatively short, at ~1,400 bp and ~ 700 bp, respectively. To test whether the prepared templates were useful for amplifying long and single copy gene fragments, we chose a DNA fragment about 6.5 kb to be amplified by primer pair ScGPD6K-F and ScGPD6K-R for *S. cerevisiae*, and MaGPD6K-F and MaGPD6K-R for *M. antarcticus*. In addition, to determine whether the method is suitable for preparing PCR template based on thick-walled fungal spores, *A. niger* conidia spores were used, and a single-copy gene (Cysteine protease gene) about 1.5 kb long was amplified by primers AgCysP-F and AgCysP-R for PCR detection. All PCR primers used in this study are shown in Table 2.

**Table 2:** Primers used in this study.

Primer name	5′ → 3′	PCR products size	Reference
F27	AGAGTTTGATCMTGGCTCAG	~1,400 bp	Córdova-Campos et al. (2012)
R1494	TACGGYTACCTTGTTACGACTT		
ITS1	TCCGTAGGTGAACCTGCGG	~700 bp	White et al. (1990)
ITS4	TCCTCCGCTTATTGATATGC		
ScGPD6K-F	TTCTACCCTTGGAATTAGTGGC	6.5 kb	This study, http://genome.jgi.doe.gov/
ScGPD6K-R	GTGAGCTCTGTAGCATTTGCAAG		
MaGPD6K-F	AGAACGGGTAGTAGATGACCACC	6.5 kb	This study, http://genome.jgi.doe.gov/
MaGPD6K-R	CCAGATCGAGGATGTTGTCTTC		
AgCysP-F	CAGTTATTCCAAGCCAGATCCC	1.5 kb	This study, http://genome.jgi.doe.gov/
AgCysP-R	GCAAGACCTTCGGACTTTGG		

### PCR and gel electrophoresis analysis

All PCR was performed in a 10 μl final reaction volume with 0.125 μM of each primer and 1 μl DNA template solution. GoTaq Green Master Mix (Promega) was used for 16S rRNA, ITS and AgCysP amplifications. Prime STAR GXL DNA Polymerase (TaKaRa) was used for the amplification of the 6.5 kb long DNA fragment, following the instructions provided by suppliers. The PCR for short fragments (16S, ITS and AgCysP) were preheated to 95°C for 2 min, followed by variable numbers of amplification cycles (denaturation at 95°C for 45 s, annealing at 56°C for 45 s, and extension at 72°C for 90s) to determine effectiveness and efficiency, with a final extension at 72°C for 5 min. PCR for the 6.5 kb fragment amplification conditions were preheated to 98°C for 25 s, followed by 35 amplification cycles (98°C for 10s, 58°Cfor 15 s, and 68°Cfor 6.5 min), with a final extension at 68°C for 10 min. Finally, 2 μl of PCR product from each amplification was used for 1% agarose gel electrophoresis and stained by GelRed.

### Integrity analysis of DNA or cells treated by PBC

To study the possible mechanisms of PBC protocol for DNA template preparation, several experiments were conducted using cells of two or three of the following species *S. cerevisiae, M. antarcticus,* and *U. maydis*. First, to investigate whether the DNA can be precipitated by PBC and to test the stability of DNA in PBC, parallel genomic DNA (gDNA) samples of *S. cerevisiae* and *M. antarcticus* were, respectively, treated by PBC (0.1 M KOH, 100°C water bath and centrifugation), “PBC-like 1” treatment (0.1 M KOH and centrifugation, without 100°C water bath) and “PBC-like 2” treatment (100°C water bath and centrifugation, without 0.1 M KOH). The centrifuged sediments and supernatants were separately detected by agarose gel electrophoresis stained by GelRed. In addition, the parallel gDNA samples extracted using commercial kits placed at room temperature (for the duration of PBC, ~25 min) and stored at-20oC were used as controls.

To investigate the effect of PBC on microbial cells, parallel cell suspensions of *S. cerevisiae*, *M. antarcticus* and *U. maydis* were treated by PBC, EtNa, and 100°C water baths without any alkaline agent (named Boiled). The final centrifuged sediments were analyzed by flow cytometry (FC) and scanning electron microscopy (SEM). Their corresponding untreated cells were used as controls (named Untreated). In FC analysis (S3e™ Cell Sorter, Bio-Rad), 100,000 particles were counted for each sample. According to the principle of flow cytometry, a low value of sidescatter (SSC) indicates a low internal structure complexity of the particle (corresponding to lysed cells), while a low value of forwardscatter (FSC) indicates a small volume of particles (corresponding to cell debris). A range “R1” was drawn based on the scatter distribution pattern, with particles falling into “R1” considered as seriously damaged cells. Among the total counted particles, the proportion of which falling into “R1” can partially represent the effect of treatment. In SEM analysis (SEM_6380LV, JEOL Ltd), samples treated by routine processing procedure were immersed in a fixation solution containing 2.5% glutaraldehyde. The fixed samples were investigated at a voltage of 20 kv. Three repeats were conducted for each treatment, and each sample was observed for at least 10 visual fields. The consistency among repeated samples in the same treatment was evaluated based on photographs taken of the visual fields.

## Results

### Development of PBC

#### Alcohol has a negative effect in one-step DNA template preparation

While conducting the “EtNa” protocol (Vingataramin and Frost, 2015), tube caps often popped open due to increased pressure within tubes during hot water bath (80°C) treatment and the low azeotropic temperature of ethanol/water mixture (78.07°C). However, higher temperature is beneficial for DNA extraction during alkali treatment. To overcome this issue, n-propanol was used instead of ethanol while other components remained unchanged. The temperature of water bath subsequently raised to 85°C which is a little lower than the azeotropic temperature of n-propanol/water (87.81°C). This adjustment greatly reduced the probability of tube caps from being spontaneously forced to open. Indeed, we observed no cap being popped open during water bath treatment when n-propanol/water was used for DNA precipitation.

**Figure 1:**
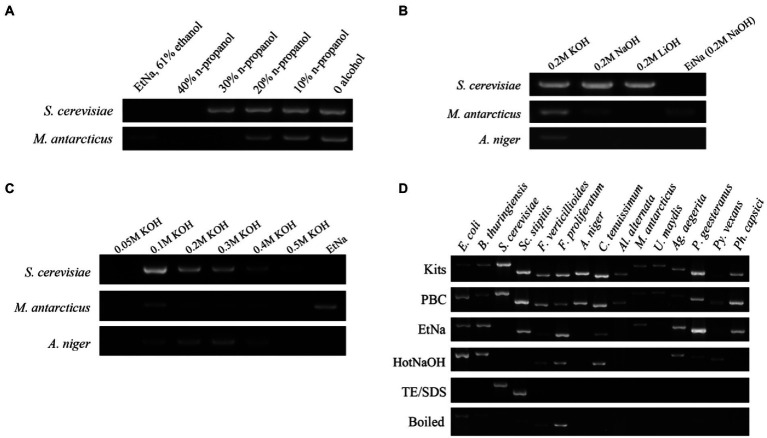
Development of the PBC technique **(A–C)** and its application **(D)**. **(A)** ITS amplification efficiency of templates prepared by NaOH solutions containing different concentrations of n-propanol. DNA template prepared by the original EtNa method (contain 61% ethanol) was used as a control, the others were prepared by a moderate modification of EtNa by replacing ethanol with n-propanol at different concentrations. Two yeasts, *S. cerevisiae* and *M. antarcticus* were used in this experiment. **(B)** ITS amplification efficiency from DNA templates prepared with different alkali reagents. KOH, NaOH and LiOH reagent solutions at the same concentration of 0.2 M were used to prepare the DNA templates for PCR from *S. cerevisiae*, *M. antarcticus* and *A. niger*. The parallel samples treated by EtNa method were used as controls. **(C)** A series of concentrations (0.05 M to 0.5 M) of KOH solutions were used to prepare the DNA templates of *S. cerevisiae*, *M. antarcticus* and *A. niger*. Parallel samples treated by the EtNa method were used for comparisons. **(D)** ITS amplification efficiency of fungal DNA templates made from a commercial DNA extraction kit, PBC and other common rapid template preparation techniques. Fifteen microbial species (including 2 species of bacteria, 2 species of oomycetes and 11 species of fungi), representing 13 families, 6 phyla and 3 kingdoms, were used for efficiency comparison of six DNA template preparation methods. All these tests for fungal species were performed using 26 cycles of PCR amplification with ITS primers.

The five gradient concentrations of n-propanol that we tested using 26 cycles of PCR showed differences in ITS amplification (Figure 1A). Specifically, in the samples treated with 40% n-propanol, neither *S. cerevisiae* nor *M. antarcticus* had visible bands. Among other treatments, when the concentration of n-propanol decreased from 30 to 0%, the bands became brighter with decreasing n-propanol concentration. The brightest amplified product was the treatment with no n-propanol. These results indicated that alcohol seemed to have a negative impact on DNA template preparation for PCR in these protocols. Thus, alcohol was excluded in our next experiment of alkali improvement.

#### Alkali agent selection and concentration optimization

EDTA is commonly used for chelating divalent metal cations such as Mg^2+^ and Ca^2+^to decrease the activities of DNA degrading enzymes and maintain DNA integrity during DNA extraction. Theoretically, a high pH solution plus boiling water (100°C) treatment should be sufficient to denature most proteins including DNA degrading enzymes such as DNase. Thus, EDTA was eliminated to further simplify the protocol in this experiment. Three alkali agents KOH, NaOH and LiOH were used to compare their effects on treatments of *S. cerevisiae*, *M. antarcticus* and *A. niger* cells. Since there was no alcohol in the treatment mixture, the water bath temperature was increased to the boiling temperature to further improve treatment effects. Using EtNa protocol as a control, the 26 cycles of PCR amplification using ITS primers with the centrifuged sediments of *S. cerevisiae*, *M. antarcticus* and *A. niger* treated by 0.2 M KOH treatment as templates were all successful (Figure 1B). In fact, the results using 0.2 M KOH treated samples were better than those treated with NaOH and LiOH. LiOH treatment had the lowest efficiency among the three alkali agents, and it was only effective for *S. cerevisiae*. In addition, in the EtNa control, only *M. antarcticus* showed a visible band, while the other two fungi had no visible band. Thus, KOH was chosen as the most suitable alkaline agent for subsequent concentration optimization experiment.

To optimize the application concentration of KOH, we set a series of KOH concentrations for the treatment mixtures, from 0.05 M to 0.5 M. These mixtures were all treated in the same 100°C water bath, centrifuged under the same condition, then the centrifuged sediments were all tested using 26 cycles of PCR amplification with ITS primers. The results are shown in Figure 1C. Compared to other KOH concentrations, the amplified DNA bands of the three fungi treated in 0.1 M KOH are all the brightest, especially for *S. cerevisiae* and *M. antarcticus*. When the concentration of KOH in mixtures increased from 0.1 M to 0.5 M, the PCR amplified bands of *S. cerevisiae* and *M. antarcticus* became weaker with the increase of KOH concentration. For *A. niger*, though the samples treated by 0.2 M and 0.3 M KOH showed brighter PCR amplification bands, the DNA template prepared by 0.1 M KOH treatment also showed an acceptable result. In contrast, there was no visible DNA band for the three fungi treated by 0.05 M KOH. Among the three fungi treated by EtNa as controls, only the *M. antarcticus* sample had a visible band, which was consistent with the previous results. Thus, the optimal concentration of KOH was determined as 0.1 M.

Based on the above results, a rapid DNA template preparation technique for PCR was developed. We named it potassium hydroxide + boiling + centrifugation (PBC). In this protocol, microbial cells were suspended in a 0.1 M KOH solution, placed in 100°C boiling water bath for 10 min, and followed by centrifugation at 13000 g for 10 min. After resuspension into ddH_2_O, the centrifuged sediments can be used as DNA template for PCR.

### The PBC protocol can be applied to a wide group of microbes

To test whether the PBC protocol is widely applicable, 15 species of microorganisms were screened, representing 13 families, 6 phyla and 3 kingdoms (2 bacteria, 2 oomycetes and 11 fungi). The specific species and strains are shown in Table 1. The cell samples of all 15 species were each divided into 6 equal portions and, respectively, extracted using 6 treatments including PBC, EtNa, HotNaOH, TE/SDS, boiled (samples directly placed in 100°C water bath) and commercial DNA extraction Kits (one for bacteria and one for fungi). DNA templates prepared by these techniques were normalized as described in detail in “Materials and methods,” and these DNA templates were tested by the 16S rRNA and the ITS primers for bacteria and fungi/oomycete, respectively, using 26 cycles. As showed in Figure 1D, DNA templates of all 15 microbial species treated by PBC were clearly detected, with the PCR amplification efficiencies of PBC-treated samples being similar to those of commercial DNA extraction kits. For the 15 microbial species samples treated by the EtNa protocol, 10 of them could be clearly detected, two (*U. maydis* and *Py. vexans*) showed very weak bands and three (*S. cerevisiae*, *A. niger* and *Al. alternata*) could not be detected. For the same microbial samples, the HotNaOH technique led to successful amplification from 8 species; the TE/SDS technique only worked on 2 species, and direct boiling at 100°C water baths obtained weak detection of 7 species. Except for the commercial kits and PBC, no technology screened in this study succeeded in preparing DNA templates from *A. niger* and *Al. alternata*. By comparing the main characteristics of these 6 methods, including chemical components, number of protocol steps (complexity), total operation time, and the range of applicable species, the PBC method shows obvious advantages over other methods (Table 3).

**Table 3:** Comparison among rapid DNA preparation methods.

Method	Composition	Number of steps (complexity)	Total operation time	Scope of application§
Commercial kit	Enzymes, 4 Buffer solutions (composition unspecified)	10	2 h	15/15
PBC	KOH	2	20 min	15/15
EtNa	NaOH, Ethanol and EDTA	2	20 min	12/15
HotNaOH	NaOH, EDTA and Tris–HCl	2	30 min	8/15
TE/SDS	SDS, EDTA, NaCl, Tris–HCl, Sodium acetate, Ethanol, and Isopropanol	9	30 min	2/15
Boiled (direct colony PCR)	-	-	-	7/15, dim

**§**Scope of application refers to the number of species successfully amplified out of 15 species investigated in this study.

### PBC is sensitive, can be used to detect a range of cell concentrations

To further investigate the sensitivity of PBC, yeast cells of *S. cerevisiae* and *M. antarcticus* cultured in liquid medium were investigated using serially diluted cells. The serially diluted cell populations at 10^8^ cells/ml, 10^7^ cells/ml, 10^5^ cells/ml, 10^3^ cells/ml and 10 cells/ml were treated by PBC, while parallel samples were treated by EtNa for comparisons. The centrifuged sediments of these two methods were normalized by dissolved in sterile water volume equivalent to the original cell suspension samples. And then 1 μl from each treatment was used for PCR amplification using the ITS primers. Specifically, there were 10^5^, 10^4^, 10^2^, ~1 and 0.01 estimated cells in the corresponding PCR reaction systems, respectively. The treatment effects between PBC and EtNa were tested by amplification with the ITS primers using 26 cycles, 30 cycles and 35 cycles of PCR, respectively.

**Figure 2:**
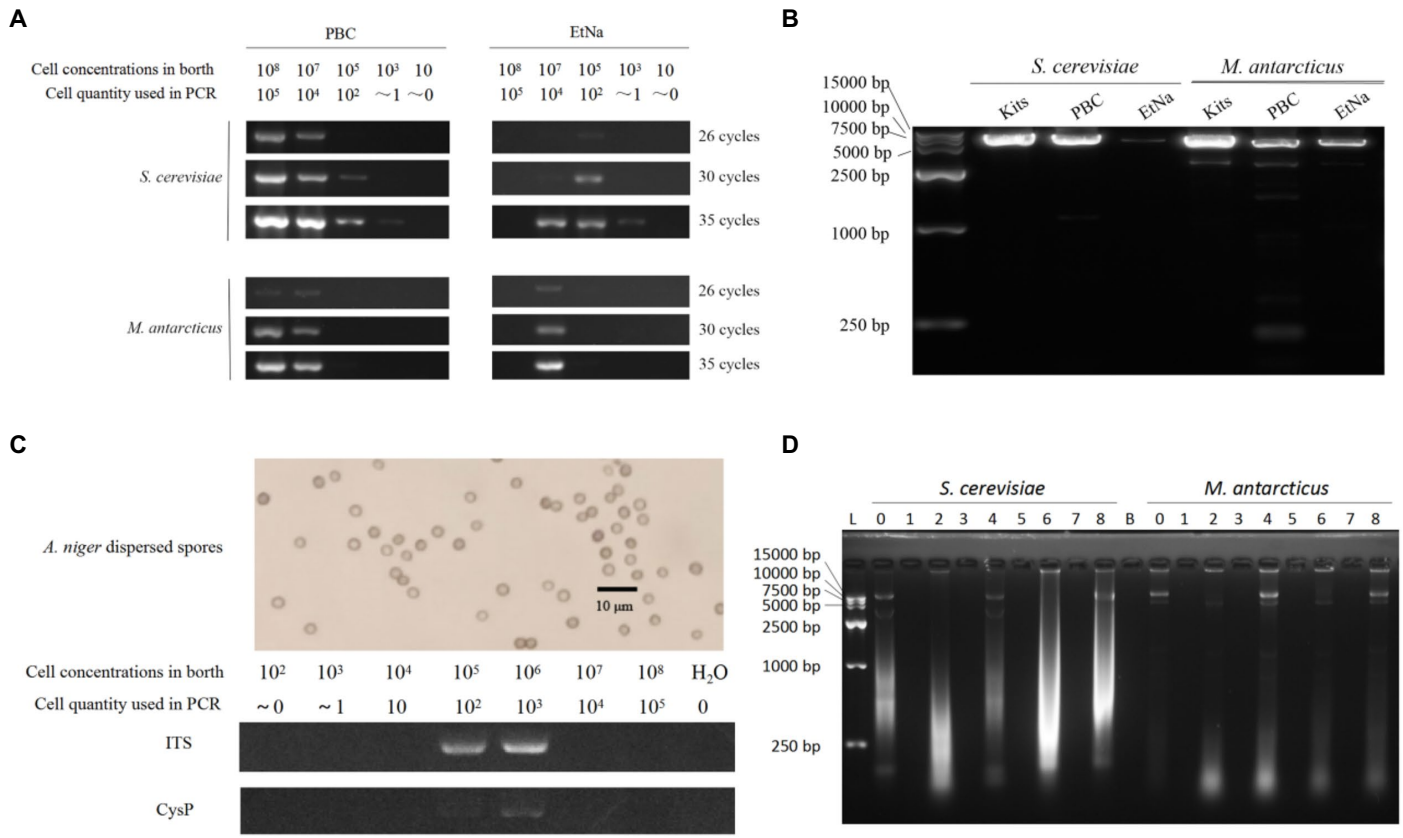
PBC applications **(A–C)** and its inability to precipitate free DNA **(D)**. **(A)** Comparison between PBC and EtNa methods for treating different cell density samples. *S. cerevisiae* and *M. antarcticus* samples of different cell densities were treated by PBC and EtNa, respectively. The templates were then tested by PCR amplification using ITS primers with 26, 30, and 35 PCR amplification cycles. **(B)** Comparing the amplification efficiencies of long DNA fragment from the templates made by commercial kits，PBC and EtNa, respectively. Single copy DNA fragments of about 6.5 kb (containing the full-length glyceraldehyde 3-phosphate dehydrogenase gene) from *S. cerevisiae* and *M. antarcticus* were used in this test. **(C)** PBC application in thick cell wall sample. Well-dispersed *A. niger* spore (above) and PCR amplification from this spore solution of different concentrations treated by PBC (bellow). **(D)** PBC is unable to precipitate free DNA. Purified DNA was treated in different ways and separated into deposits and supernatants by centrifugation. Both deposits and supernatants were checked by agarose gel electrophoresis, respectively. Lane 0: DNA stored at −20°C. Lane 1: the centrifuged sediments from PBC (0.1 M KOH, boiling and centrifugation) treatment. Lane 2: the supernatant from PBC treatment. Lane 3: the centrifuged sediment from “PBC-like 1” treatment (without boiling in 100°C water bath). Lane 4: the supernatant from “PBC-like 1” treatment (without boiling in 100°C water bath). Lane 5: the centrifuged sediment from “PBC-like 2” treatment (without KOH). Lane 6: the supernatant from “PBC-like 2” treatment (without KOH). Lane 7: the centrifuged sediment of DNA solution kept at room temperature for the same period and centrifuged. Lane 8: the supernatant of DNA solution kept at room temperature for the same period and centrifuged. Lane B: blank.

The detection results are shown in Figure 2A. For *S. cerevisiae* treated by PBC, the samples with concentrations from 10^3^ cells/ml to 10^8^ cells/ml were detected by after 35 PCR cycles. Specifically, the sample of 10^3^ cells/ml concentration (corresponding to one cell in the PCR reaction) showed a faint band while the samples of 10^8^ cells/ml and 10^7^ cells/ml showed very bright bands. Among the parallel samples treated by EtNa, those from 10^3^ cells/ml to 10^7^ cells/ml of *S. cerevisiae* showed weak bands after 35 PCR cycles. For *M. antarcticus*, the samples of 10^8^ cells/ml and 10^7^ cells/ml treated by PBC were clearly detectable but only the 10^7^ cells/ml sample treated by EtNa was detectable after 35 PCR cycles. As the number of PCR cycles decreased, the brightness of the amplified bands gradually decreased. The results of this experiment indicated that PBC performed better than the EtNa protocol for different cell densities of samples.

### PBC is applicable for long fragment amplification

In general, it is difficult to amplify long DNA fragments from poor quality DNA template. To verify whether the DNA template made by PBC is suitable for amplifying long DNA fragment, two gene fragments (containing glyceraldehyde 3-phosphate dehydrogenase gene) of 6,493 bp and 6,504 bp from *S. cerevisiae* and *M. antarcticus, respectively,* were used in this experiment. Both fragments are single copy in their genomes (querying in JGI database^2^).The templates were amplified by 35 cycles of PCR, and the results are shown in Figure 2B. The PCR amplified DNA band of the *S. cerevisiae* sample made by PBC was very bright, close to the efficiency of templates made using the commercial kit and brighter than that using the EtNa method. For the *M. antarcticus* sample, the PCR amplified DNA band of PBC was brighter than that of EtNa, but weaker than that prepared using the commercial kit. Overall, the quality of DNA templates prepared by PBC is sufficient for the amplification of long DNA fragments.

### PBC is applicable to samples with thick cell walls

As a common fungal reproductive organ, spores usually have very thick cell walls and are resistant to a variety of environmental stresses. Therefore, fungal spores are usually more difficult to break (Jones, 2006; Dijksterhuis, 2019) and to extract DNA from than fast-growing vegetative mycelia. To investigate the effectiveness of PBC for preparing fungal spores for PCR, a range of concentration of *A. niger* spores from 10^2^ to10^8^ were used in this experiment. After treated by PBC (10 min in 100°C water bath) and 35 cycles of PCR amplifications of the ITS and Cysteine Protease gene (CysP，single copy about 1.5 kb long) fragments, the samples of 10^5^ cells/ml and 10^6^ cells/ml showed clear ITS bands. However, only the 10^6^ cells/ml sample showed a weak band for the CysP fragment, as shown in Figure 2C. There was no visible band in samples with other spore concentrations. These results indicate that PBC is applicable for preparing DNA template from fungal spore samples at 100–1000 spores per reaction.

### PBC is applicable for high throughput analyses

**Figure 3:**
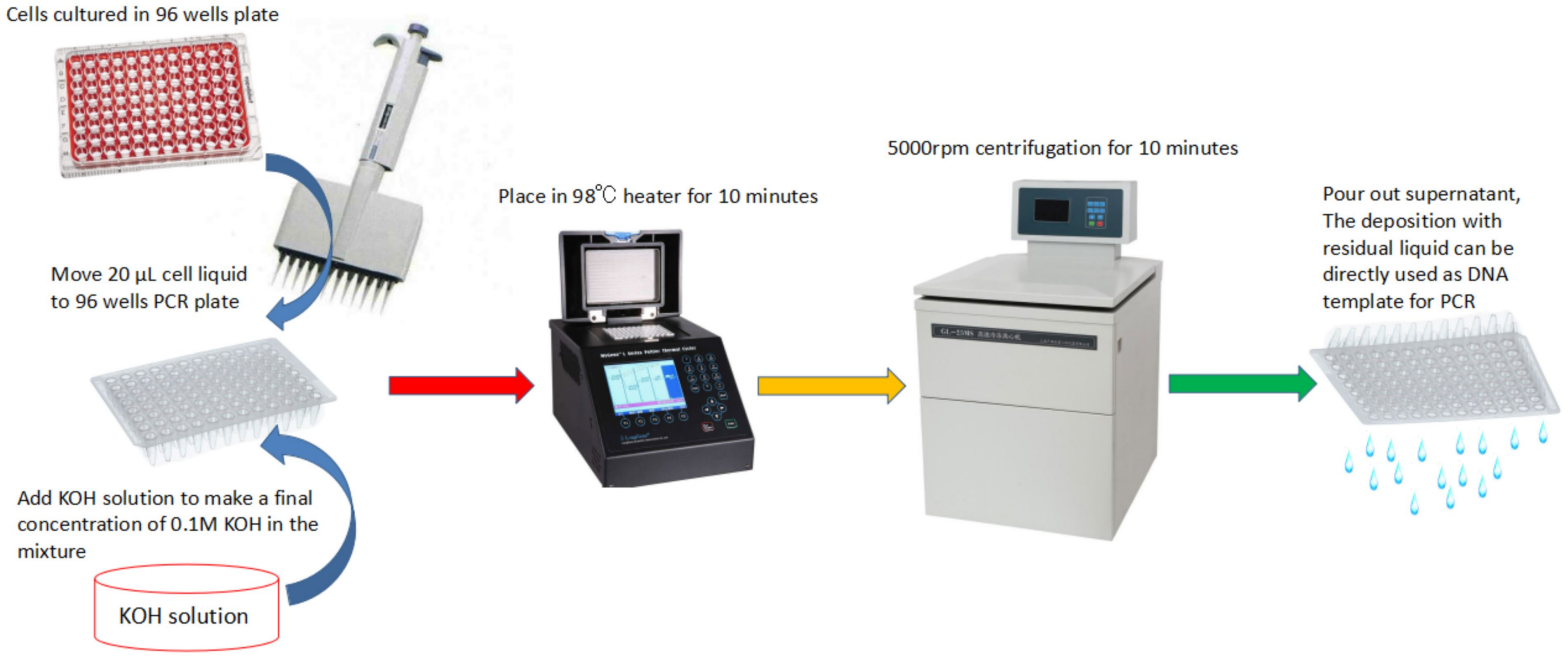
The PBC technique in a high throughput format for a large number of samples.

As shown in Figure 3, a 96-well PCR plate can be used in the PBC protocol for high throughput analyzes. In the high throughput format, cell suspensions and KOH solution were mixed in each well to reach a final KOH concentration of 0.1 M, then the plate was heated in a PCR instrument at a temperature of 98°C for about 10 min (or appropriately extended the time for thick cell wall sample), and then centrifugated for about 10 min in 5,000 rpm rotation. After taking out the supernatant, the residual liquid and cell sediment (or alternatively diluted deposition with ddH_2_O) can be directly used as DNA templates for PCR. Although the residual liquid still has a high pH (about pH ≈ 9.0), the small amount (about 1 μl) used in each PCR is not enough to change the pH value of PCR reaction buffer system. Such high throughput templates can completely satisfy the general requirement of PCR amplification. We have already used this technique to make PCR templates from hundreds of transgenic clones of *U. maydis* within half an hour and successfully detected the target insert or deleted DNA fragment among tested samples (data not shown).

### The possible mechanisms of PBC

#### PBC cannot precipitate free DNA in solution

In almost all DNA extraction protocols, alcohol is used as a DNA precipitant to separate DNA from water by breaking the hydrogen bond between the two, which alkali cannot do. However, our results shown in Figure 1A seemed to suggest that DNA templates could be precipitated even without alcohol. Consequently, to investigate whether KOH alone could precipitate DNA, purified genomic DNA samples (extracted by commercial kits) of *S. cerevisiae* and *M. antarcticus* were treated by PBC, “PBC-like 1” and “PBC-like 2” treatments, respectively. Then the centrifuged sediments and supernatants were checked by agarose gel electrophoresis stained with GelRed at a sensitivity detection limit of 0.5 ng DNA (Haines et al., 2015). As shown in Figure 2D, there was no band in any of the centrifuged precipitates (Lanes 1, 3, 5 and 7), regardless of whether the treated mixture contained KOH or not. This result suggested that KOH could not precipitate DNA. The results also showed very low quantity of DNA when known extracted DNA samples were further treated in 100°C water baths, especially when KOH was present. This experiment indicated that PBC treatment cannot precipitate free DNA in solution but likely cause damage to cells.

#### PBC damages cells and makes DNA available for PCR

In the PBC products of *S. cerevisiae* and *M. antarcticus* cells, there was no visible or smeared DNA band detectable by agarose gel electrophoresis. In addition, Nanodrop readings showed very low DNA concentration (<10 ng/μL) (data not shown). The low DNA concentration reading of Nanodrop is typically unreliable because of impurity or contamination by other cellular components. In addition, the free DNA cannot be precipitated during PBC treatment and can even be degraded during this treatment, thus the DNA templates made by PBC were unlikely free DNA. To determine the status of DNA from PBC treatment, the centrifuged sediments of *S. cerevisiae*, *M. antarcticus* and *U. maydis* treated by PBC, EtNa and 100°C water bath were used for FC analysis and SEM observations.

**Figure 4:**
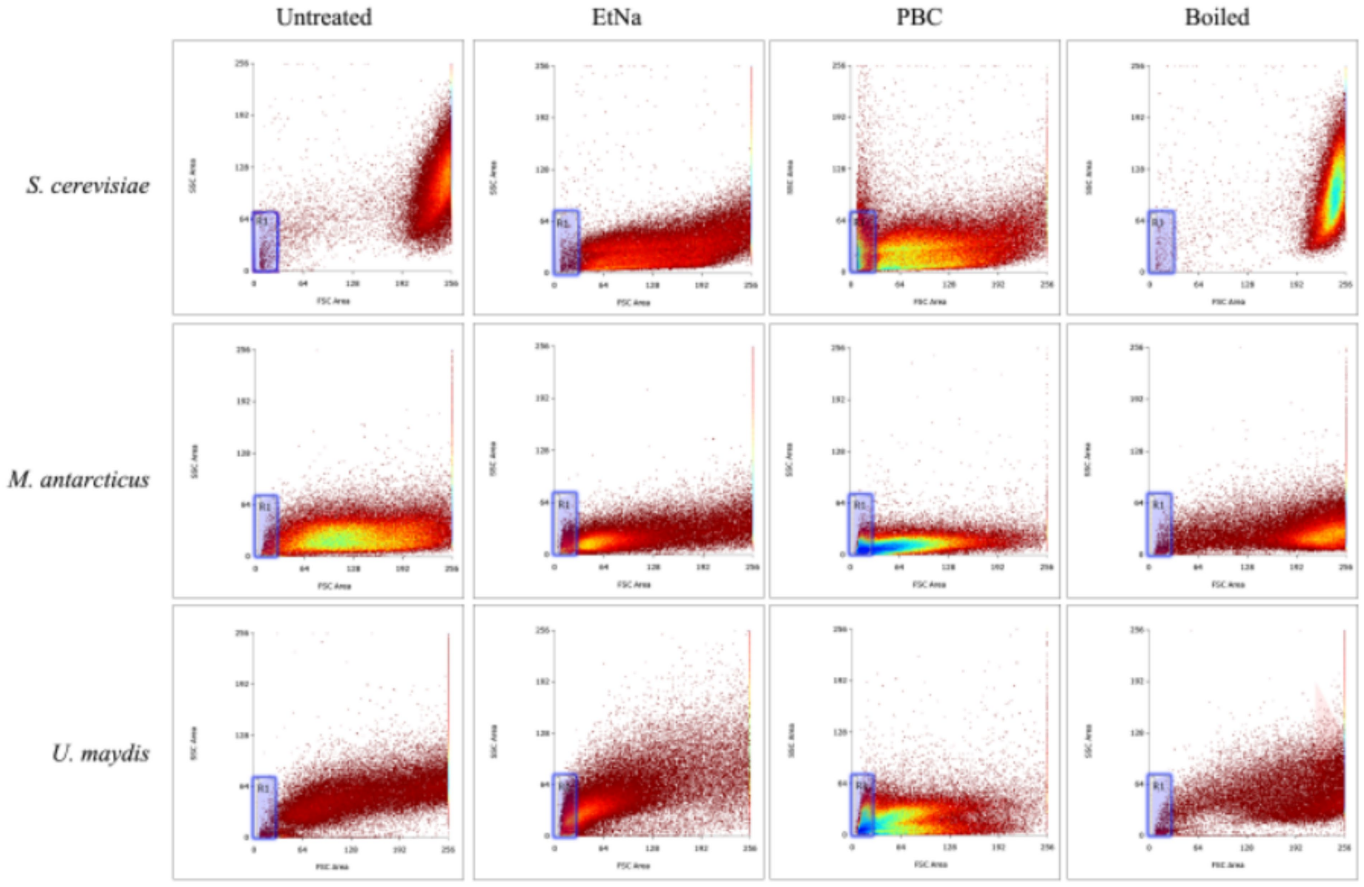
Flow cytometry analysis of microbial cells with different treatments. Flow cytometry was used to detect the cell states of *S. cerevisiae*, *M. antarcticus* and *U. maydis* after 100°C water bath (boiled), PBC and EtNa treatments, respectively. Untreated cells were used as control. The “R1” area is marked with a blue frame.

The results of FC analysis are shown in Figure 4. Compared with the boiled and the untreated samples, the centrifuged sediments of *S. cerevisiae*, *M. antarcticus* and *U. maydis* treated by PBC had more particles fell into the “R1” area, and the total counted particles had lower values of SSC and FSC. For the *S. cerevisiae* samples treated by EtNa, though the total counted particles had lower values of SSC and FSC than those of boiled and untreated samples, there were fewer particles fell into the “R1” area. In contrast, there were more particles of *M. antarcticus* and *U. maydis* treated by EtNa fell into the “R1” area. The percentage of particles in the “R1” area of each sample is shown in Table 4. For *S. cerevisiae*, only 0.83% particles treated by EtNa fell into “R1,” which is much closer to that of untreated and boiled samples. However, the percentage of *S. cerevisiae* particles treated by PBC in the “R1” area was higher, reaching 13.47%. For samples of *M. antarcticus* and *U. maydis*, compared with those treated by the EtNa method, the samples treated with PBC also had significantly more particles fell into the “R1” area. The percentages of particles in the “R1” area were consistent with the brightness of DNA bands in PCR analysis results described above.

**Table 4:** The percentages (%) of particles falling into the “R1” area for different species-method combinations.

	Untreated	EtNa	PBC	Boiled
*S. cerevisiae*	0.56 ± 0.09	0.83 ± 0.08	13.47 ± 0.61	0.20 ± 0.02
*M. antarcticus*	2.22 ± 0.11	8.18 ± 0.63	22.23 ± 0.36	1.58 ± 0.13
*U. maydis*	2.67 ± 0.24	15.71 ± 0.41	28.16 ± 0.79	1.98 ± 0.19

**Figure 5:**
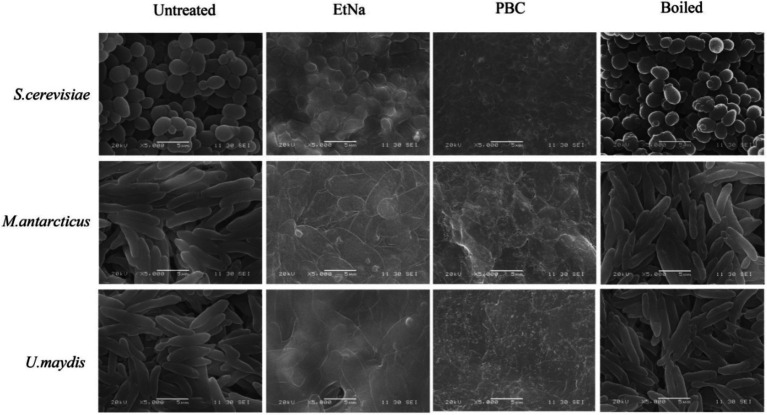
Scanning electron microscopy analysis of cell states after different treatments. Scan images show cells of *S. cerevisiae*, *M. antarcticus* and *U. maydis* after 100°C water bath (boiled), PBC and EtNa treatments. Untreated cells were used as controls.

Results of SEM observations of centrifuged sediments are shown in Figure 5. The untreated cells were round and had intact thick cell wall, while most of the boiled cells were wrinkled and hollow but still retained thick cell wall. In contrast, the cells treated by EtNa were flat (except *S. cerevisiae*) and had thin cell wall. However, most of them retained intact cell shape. Compared with cells treated by EtNa, those from PBC treatment were severely damaged, most of them had no complete cell structure. These SEM results were also consistent with those of FC analysis (Figure 4) and PCR amplification results (Figure 1D).

Together, these analyzes indicated that DNA templates made by PBC were not naked free DNA but were long DNA molecules inside damaged cells easily accessible by the PCR system. In addition, samples prepared by the PBC method were stable and similarly suitable for PCR after storage at both room temperature and in 4°C refrigerator for at least overnight [data not shown].

## Discussion and conclusion

Simple and rapid technique for preparing DNA template for PCR is very useful in biological research, medical examination and other applications based on PCR. In this study, we developed a PBC technique for PCR template preparation, with the characteristics of low cost, fast processing speed, wide application for diverse cell samples and high throughput compatibility. There have been many studies on rapid DNA extraction techniques in the last few decades, including several that use hot sodium hydroxide. However, in protocols using NaOH, the concentration of NaOH is typically low (bellow 50 mM). Such a low concentration of NaOH may have a limited effect on cells with a thick cell wall and thus the protocols are not broadly applicable (Rudbeck and Dissing, 1998; Truett et al., 2000). The EtNa method was a bold approach to use NaOH at a high concentration of 0.2 M together with ethanol (as a DNA precipitant). However, at the same concentration, KOH was found to be more effective than NaOH in DNA template preparation (Figure 1B). In histological studies, KOH also showed better effects on separating tissues than NaOH (Das and Barringer, 2006; Max et al., 2009), even when the concentration of KOH is much lower than that of NaOH. Thus, alkalinity itself does not seem to fully explain this phenomenon that KOH is more potent than NaOH. Additional mechanism may be involved. For example, KOH and NaOH are known to differ in their interactions with carbon-based materials, and K^+^ has a stronger tissue penetration effect than Na^+^ during the interactions (Raymundo-Pinero et al., 2005). Therefore, we believe that K^+^ plays a major role in the effectiveness of PBC for PCR template preparation. Aside from the greater efficiency, PBC also worked on a broader range of microbial species (Figure 1D) and cell concentrations (Figure 2A) than other rapid DNA template preparation techniques.

To solve the EtNa protocol problem of unwanted cap popping, we accidentally found that ethanol may act negatively during alkali treatment, as did n-propanol (Figure 1A). So, alcohol is not necessary for rapid DNA template preparation in our one step treatment. We speculated that “DNA templates” that were centrifuged and precipitated without any alcohol (Figure 1A) were not naked free DNA but were bound within damaged cells. Indeed, naked free DNA could not be precipitated in PBC treatment and were likely degraded in such a treatment (Figure 2D). FC analysis showed that there was a higher proportion of cell debris in the centrifuged sediment from PBC treatment than other treatments. SEM observations further confirmed that there were more damaged cells or cell debris in PBC products than in other treatments. Our results indicated that the higher content of cell debris and damaged cells in the centrifuged sediments, the brighter the DNA band from PCR amplification. It is well-known that cells with damaged structures can be directly used as DNA templates for PCR. Thus, we deduced that the products of PBC were not naked free DNA but were structurally damaged cells or cell debris. This is a key principle of alkali rapid DNA template preparation techniques that has not been mentioned before.

Together, PCR template preparation based on PBC is essentially different from the concept of “DNA extraction,” which aims to gain purified free DNA. It is also different from the technique of rapid nucleic acid purification by cellulose-based paper (in 30 s; Zou et al., 2017), which aims to capture free DNA and concentrate the DNA from the sample solution. For the cellulose-based paper technique, there is still the main technical bottleneck of releasing the captured DNA associated with biomaterials such as cell walls. In our study, PBC happens to overcome this technical bottleneck to damage cells. Although there may not be free DNA in the final products, the structurally damaged cells are sufficient as template for PCR. Skipping the extraction and purification of free DNA, PBC can be a simpler and more convenient method for rapid preparation of DNA template for PCR. One caveat of the PBC method is that it may require different cell concentrations for different microorganisms or different types of cells. If the cell concentration is higher or lower than a certain amount, the specific microorganism may not be detected by PCR (Figures 2A,C). Thus, pilot experiments may be needed in order to determine the most appropriate cell concentration for PCR template preparation for specific organisms and cell types. For samples with extremely thick cell walls, reducing cell concentration and/or extending water bath time of PBC were found to improve PCR detection effect (data not shown).

In this study, we used pure microbial cultures to develop the PBC method and test its effectiveness. Whether the PBC method will work for complex environmental samples remains to be determined. Our preliminary results with diseased plant leaves showed that the PBC technique can be used to prepare DNA template of pathogens for PCR. Specifically, using the PBC technique to treat diseased leaves of Huang Jing (*Rhizoma polygonatum*) and soybean (*Glycine max*), followed by PCR using ITS primers and sequencing of the amplified products, we were able to identify *Fusarium avenaceum* as the causative agent of infection in a Huang Jing leaf, and three fungal pathogens (*Diaporthe phaseolum*, *Colletotrichum fructicola* and *Fusarium irregulare*) infecting a soybean leaf (data not shown). However, we did not compare our PBC method with existing methods for its efficiency and sensitivity for the leaf samples. A comprehensive analysis would require metabarcode sequencing of PCR-amplified products and/or metagenome shotgun sequencing of the total environmental DNA for species identification and whole metagenome data mining (e.g., Xu, 2016, 2020). For more complex samples such as soil or compost, additional treatments may be needed to remove potentially PCR-inhibitory substances from the samples before PBC treatment. Empirical tests are required to identify the inhibitory substances within each type of samples and the methods to remove them. While we believe such analyzes could be very interesting, they are beyond the scope of this paper.

In summary, the PBC protocol is broadly applicable for rapid DNA template preparation for PCR. It’s simple, fast, and inexpensive and can be used for a diversity of microorganisms for amplifying both short and long fragments in a high throughput format. Although it has not been tested in this study, PBC could be adapted for rapid preparation of DNA templates from animal, plant and environmental samples for PCR. We hope that the PBC technique will facilitate PCR-related biological research.

## Data availability statement

The raw data supporting the conclusions of this article will be made available by the authors, without undue reservation.

## Author contributions

YLiu and JC conducted the experiments and contributed equally to this work. ZL and JX conceived and designed the experiments. ZL and YLiu wrote the draft. XX and YLin contributed to microbial species selection and identification. YLi, XL, ZZ, YC, JC, and JX revised the manuscript. All authors contributed to the article and approved the submitted version.

## Funding

This research was financially supported by the Central Public-Interest Scientific Institution Based Research Fund (no. 1610242016030), Scientific Research Project of Hunan Plant Protection and Inspection Station for plant epidemic prevention and control (HNZB202104), and Agricultural Science and Technology Innovation Program (no. ASTIP-IBFC-04). This research was supported by equipment provided by Key Laboratory of Biological Processing of Bast Fiber Crops, MOAR.

## Conflict of interest

The authors declare that the research was conducted in the absence of any commercial or financial relationships that could be construed as a potential conflict of interest.

The reviewer AC declared a shared affiliation with the authors YL, JC, YC, and ZL to the handling editor at the time of review.

## Publisher’s note

All claims expressed in this article are solely those of the authors and do not necessarily represent those of their affiliated organizations, or those of the publisher, the editors and the reviewers. Any product that may be evaluated in this article, or claim that may be made by its manufacturer, is not guaranteed or endorsed by the publisher.

## Footnotes

^1^ http://vle-calc.com/index.html

^2^ http://genome.jgi.doe.gov/
